# Untangling Impacts of Socioeconomic Position, Chronic Disease, and Low-Level PM2.5 Exposure on Mortality Among Native American Medicare Beneficiaries

**DOI:** 10.3390/ijerph23040464

**Published:** 2026-04-04

**Authors:** Judy Wendt Hess, Wenyaw Chan

**Affiliations:** 1 Hess Epidemiology Services, LLC, Houston, TX 77018, USA; 2 Department of Biostatistics and Data Science, University of Texas Health Science Center at Houston, Houston, TX 77030, USA; wenyaw.chan@uth.tmc.edu

**Keywords:** American Indian or Alaska Native, mortality, fine particulate matter, socioeconomic position, chronic disease, principal components analysis, Medicare, tribal lands

## Abstract

**Highlights:**

**Public health relevance—How does this work relate to a public health issue?**

- Long-term exposure to fine particulate matter (PM2.5) has been linked to increased mortality, including at concentrations below current regulatory standards.
- We evaluated whether the association between low-level PM2.5 exposure and all-cause mortality observed in the Medicare cohort is broadly generalizable or influenced by a geographic clustering of sociodemographic characteristics and underlying disease burden.

**Public health significance—Why is this work of significance to public health?**

- Our results suggest that the PM2.5-related mortality increase observed among Native American beneficiaries was influenced by geographic clustering in a small number of western states.
- Adjustment for state-level context, baseline health status, and area-level socioeconomic conditions largely attenuated the PM2.5–mortality association in this low-exposure population.

**Public health implications—What are the key implications or messages for practitioners, policy makers, and/or researchers in public health?**

- Associations between PM2.5 and mortality observed at very low exposure levels should be interpreted with careful consideration of geographic composition, socioeconomic conditions, and baseline health characteristics within large administrative cohorts.
- Further research is needed to confirm whether mortality associations observed at low PM2.5 levels reflect causal exposure effects or characteristics of the populations represented in low-exposure environments.

**Abstract:**

Ambient fine particulate matter (PM2.5) is associated with increased mortality at concentrations below current regulatory standards. Studies of low-level exposure often rely on large administrative cohorts whose geographic and demographic composition may influence observed associations. In a prior analysis, we observed an association between long-term PM2.5 and all-cause mortality among Native American Medicare beneficiaries living in zip codes within the lowest decile of PM2.5 exposure. The present study, a case–control analysis of 1,713,399 low-PM2.5-exposed beneficiaries enrolled in traditional Medicare during 2015–2016, evaluated whether this association could be explained by geographic context, socioeconomic position (SEP), or baseline health status. We used principal components analysis to summarize area-level SEP indicators and beneficiary-level chronic disease diagnoses. In fully adjusted pooled models, PM2.5 was more strongly associated with mortality among Native American beneficiaries (odds ratio, OR = 1.12 per ug/m^3^; 95% CI 1.06–1.18) than among non-Native American beneficiaries (OR = 1.01 per ug/m^3^; 95% CI 1.001–1.02). Sequential adjustment among Native Americans showed that state-level geographic clustering accounted for most attenuation of the PM2.5 coefficient, with additional modest attenuation after adjustment for SEP and chronic disease patterns. These findings suggest that PM2.5–mortality associations observed in low-exposure populations may partly reflect geographic composition and underlying health differences within these large cohorts.

## 1. Introduction

Ambient fine particulate matter (PM2.5) is a well-recognized environmental health risk, leading to an estimated 70,000 deaths per year in the U.S. [1]. Associations between long-term PM2.5 exposure and all-cause mortality have been demonstrated in numerous studies across diverse populations, geographic settings, and exposure concentrations [2,3,4]. This includes a body of literature reporting mortality effects at concentrations well below the current U.S. Environmental Protection Agency (EPA) National Ambient Air Quality Standard (NAAQS), with little evidence of a threshold and, in some analyses, steeper slopes at lower concentrations [5,6,7,8]. These low-exposure analyses have relied upon large administrative cohorts in which demographic groups, health characteristics, and burden of disease—as well as ambient PM2.5 concentrations—are unevenly distributed across geographic regions. Whether the reported low-level associations reflect broadly generalizable exposure-response effects or a result of geographic clustering within a cohort remains unclear.

Vulnerability to PM2.5 may vary across populations due to differences in socioeconomic factors, baseline health status, healthcare access, and geographic context [9,10,11]; for example, a population with high cardiovascular and lung disease prevalence may be more susceptible to air pollution exposure, even at relatively low ambient concentrations [12,13]. Persistent disparities can shape both the distribution of chronic disease and patterns of air pollution exposure, and disentangling their independent and combined effects on mortality is complex [9,14]. Socioeconomic position (SEP) and disease prevalence may act as conventional confounders—that is, variables associated with both exposure and outcome that distort the true exposure–response relationship if not adjusted for—or as indicators of structural population-level vulnerability [10,15].

In a prior analysis of Medicare beneficiaries, we observed a slight increase in all-cause mortality (odds ratio [OR], 1.013; 95% confidence interval [CI], 1.005–1.022) per 1 µg/m^3^ increase in PM2.5 among those living within the lowest PM2.5 exposure decile [16]. In race-stratified analyses, the association persisted only among Native American beneficiaries despite having lower average PM2.5 exposure than any other racial group. An additional finding was that socioeconomic status was a substantially stronger predictor of mortality than PM2.5 across all exposure deciles. These findings raised the possibility that differences in SEP or baseline health characteristics that vary with PM2.5 at the zip code level might explain the observed heterogeneity by race.

The objective of this study was to evaluate whether our previously observed low-level PM2.5–all-cause mortality association reflects an exposure effect generalizable beyond the Medicare cohort or is influenced by the demographic, geographic, and baseline health composition of the segment of this cohort living in low-exposed zip codes, particularly the concentration of Native American beneficiaries. We restricted analyses to beneficiaries residing in zip codes within the lowest PM2.5 exposure decile and adjusted sequentially for geographic context variables, SEP, and chronic disease diagnoses to assess the extent to which these factors explained the observed racial heterogeneity. We hypothesized that adjusting for these factors would attenuate the PM2.5–mortality association previously observed among Native American Medicare beneficiaries.

## 2. Materials and Methods

### 2.1. Data Sources

#### 2.1.1. Medicare Data

This study used Medicare data obtained from the Centers for Medicare and Medicaid Services (CMS), including the Master Beneficiary Summary File (MBSF) Base and MBSF 27 Chronic Conditions Segment annual files for 2015 and 2016. The files included records of all Medicare beneficiaries 65 years of age or older living in the contiguous U.S. and enrolled in a traditional fee-for-service Medicare program for the entire calendar year. Person-level data elements in the files included age, sex, race, residential state and zip code, dual Medicare/Medicaid eligibility, history of chronic health conditions, and date of death. Beneficiaries eligible for Medicaid benefits during any month of the study period were considered dual-eligible in the analysis. The chronic conditions segment contained a history of past or current diagnoses of 27 conditions based on medical encounter records. The Medicare cohort comprises 95% of those aged 65 and older in the U.S. and accounts for 66% of deaths nationwide [6]. Low-income elderly are eligible for concurrent Medicare/Medicaid benefits, although specific eligibility rules and benefits vary by state and change over time [17]. The ‘dual’ enrollment is the only person-level SES indicator available in the MBSFs.

#### 2.1.2. Ambient Air Pollution Data

Daily average concentrations of ambient PM2.5 were obtained from the U.S. National Aeronautics and Space Administration Socioeconomic Data and Applications Center for 2014–2016 [18]. Methods used to derive these estimates have been described in detail elsewhere [19]. In brief, pollutant concentrations were predicted at the centroid of 1 km by 1 km grid cells across the contiguous U.S. using an ensemble of machine-learning models integrating more than 100 predictor variables from ground-based and satellite observations, meteorological data, land-use variables, and outputs from chemical transport models. Cross-validated R^2^, representing agreement between monitored and predicted PM2.5 values, was 0.86 across the U.S. for the period 2000–2015. Model performance in the latter part of this period was slightly lower (0.783 in 2015), and agreement also varied by region of the U.S. [19]. We used the grid cell concentration closest to the centroid of each zip code to estimate daily exposure at the zip code level on each day of 2014, 2015, and 2016. These daily pollutant estimates were then used to calculate—for each day of the study period—average exposure in the preceding 365 days (i.e., average annual exposure) for each zip code represented in the Medicare beneficiary files. From these values, and based on the full 2-year distribution, we assigned a decile of annual average PM2.5 to each zip code on each day of 2015 and 2016.

#### 2.1.3. Socioeconomic Position Data

Data elements from the 2015 Agency for Healthcare Research and Quality (AHRQ) Social Determinants of Health (SDOH) Database were used to assign zip code-level indicators of SEP [20]. Data were compiled by the AHRQ from the U.S. Census Bureau American Community Survey. SDOH indicators were retained for principal components analysis (PCA) if they were conceptually aligned with SEP, that is, those reflecting economic stability, educational attainment, housing quality and affordability, access to transportation, financial access to medical care, and social context and household structure [9]. We excluded indicators of demographic composition, health behaviors or outcomes, immigration or spoken language, geographic context (e.g., urbanicity), and those that were subgroup-specific (e.g., poverty among children) or stratified versions of a broader indicator (e.g., households receiving food stamp benefits). A full list of SDOH variables used in our analysis, grouped by SEP domain, can be found in Table S1.

#### 2.1.4. Covariate Data

Rural–urban continuum classification for each zip code was obtained from the USDA Economic Research Service 2010 Rural-Urban Commuting Area (RUCA) Codes [21]. RUCA primary code ranged from 1 (urban) to 10 (rural) and was modeled as a continuous variable with “1” as the referent value. For purposes of comparing urbanicity between subgroups, primary RUCA codes were recoded to the following categories: metropolitan (values 1–3), micropolitan (values 4–6), small town (values 7–9), or rural (value 10). The percent of Native American residents by zip code was obtained from the U.S. Census Bureau 2010 Decennial Census as an indicator of the relative concentration of Native American populations within a defined geographic area [22]. The latter was included as a community context variable in attenuation analysis restricted to Native American beneficiaries, but not in pooled or race-stratified models to avoid overadjustment in models containing person-level race. Finally, from the AHRQ SDOH database, we retained a zip code-level “tribal land” indicator, defined by the U.S. Census Bureau as a geographic area contained within an American Indian reservation or off-reservation trust lands [20,23]. This variable was not included in principal components analysis.

### 2.2. Statistical Analysis

This was a case–control study. All beneficiaries who died in 2015 or 2016 were selected as cases and assigned a decile of annual average PM2.5 exposure based on their zip codes and dates of death as described above. Up to 10 controls were randomly selected for each case from among all beneficiaries alive on the case’s date of death, matched on decile of exposure at the control’s zip code on the case’s date of death. Since roughly 10% of beneficiaries died during the study period, a ratio of 10 controls for each case allowed us to balance maximizing the number of controls per case while also ensuring that there were 10 (or close to 10) controls available for each case. Beneficiaries were excluded if their zip code was either missing or not contained in the PM2.5 files, as a decile of exposure could not be assigned. Because our analysis focused on low-exposure beneficiaries, only cases and controls in the first annual average PM2.5 exposure decile (range: 0.01–4.68 µg/m^3^) were included. Matching on PM2.5 decile was intended to ensure a sufficient sample of controls in the low exposure group, with an adequate gradient of PM2.5 exposure, while also allowing for examination of the independent impact of other covariates, including age and sex. MBSF race (Native American vs. non-Native American) originated from U.S. Social Security Administration records, based on voluntary self-reporting.

#### 2.2.1. Principal Components Analysis

PCA is a statistical method that reduces a large set of correlated variables-in this case, 34 SEP indicators and 27 chronic condition indicators-into a smaller number of underlying constructs by grouping variables according to shared covariance [24]. By consolidating highly correlated indicators, we were able to incorporate a broad set of conceptually important measures while ensuring that the resulting components represented distinct constructs, thus avoiding multicollinearity. PCA was performed separately on SEP and chronic conditions indicators and included only the lowest-decile zip codes in order to capture constructs specific to low PM2.5-exposed areas. We evaluated a combination of eigenvalues, scree plots, and the interpretation of factor loadings to determine the number of components to retain for logistic regression models.

Prior to conducting PCA, each SEP indicator was standardized to mean = 0 and standard deviation = 1 to ensure comparability of indicators with different numerical scales. Indicators were then reverse-coded where necessary so that higher values consistently indicated poorer SEP (Table S1). The first principal component derived from the SEP PCA explained the largest proportion of variance and captured core socioeconomic disadvantage/material deprivation, with strong loadings related to income, poverty, unemployment, educational attainment, public assistance, insurance coverage, and household crowding (Table S2). The second component represented housing quality/affordability and transportation, with factors related to housing cost burden and vehicle access. The third component represented household composition and occupancy, with strong loadings related to household size and housing vacancy.

The chronic condition PCA identified a primary component reflecting cardiometabolic morbidity, with high factor loadings for ischemic heart disease (IHD), congestive heart failure (CHF), chronic kidney disease (CKD), hypertension (HTN), hyperlipidemia, anemia, chronic obstructive pulmonary disease (COPD), atrial fibrillation, and diabetes (Table S3). A second component reflected cognitive decline, with high loadings for dementia. A third component represented chronic conditions associated with aging and longer-term survivorship, such as arthritis, cataracts, breast cancer, and hypothyroidism, while a fourth component summarized pulmonary disease with high loadings for COPD, lung cancer, and asthma. The fifth component summarized cancer-related conditions, with a high positive loading for prostate cancer and negative loadings for female cancers.

#### 2.2.2. Logistic Regression Attenuation Analysis

Unconditional logistic regression was used to estimate the association between annual average PM2.5 exposure (per 1 µg/m^3^ increase) and all-cause mortality among beneficiaries residing in decile 1 zip codes.

We first fit a fully adjusted pooled interaction model to evaluate effect modification by Native American status (Appendix A, Equation (A1)). Odds ratios (OR) for Native American and non-Native American beneficiaries were derived by exponentiating the linear combination of regression coefficients from the pooled model. Models stratified by Native American status were then fit to evaluate consistency with the race-specific odds ratios from the pooled model (Appendix A, Equation (A2)).

To assess the extent to which geographic, socioeconomic, baseline morbidity, and community composition variables might attenuate the observed PM2.5–mortality association among Native American beneficiaries, we fit a sequential series of increasingly adjusted models (Appendix A, Equations (A3)–(A9)): Model 1 (minimally adjusted: PM2.5 + age + sex, Equation (A3)), Model 2 (+RUCA, Equation (A4)), Model 3 (+State, Equation (A5)), Model 4 (+area-level SEP, Equation (A6)), Model 5 (+individual SEP, Equation (A7)), Model 6 (+baseline morbidity, Equation (A8)), Model 7 (+community context, Equation (A9)). Percent attenuation of the PM2.5 regression coefficient was calculated on the log-odds scale for each model in the series using the change in PM2.5 coefficient from the base model (Equation (1)).

(1) %attenuation=((βbase−βcurrent)÷βbase)×100

Analyses were performed using SAS 9.4 and SAS Enterprise Guide software (SAS Institute, Inc., Cary, NC, USA). The study was approved by the Centers for Medicare and Medicaid Services Privacy Board and the University of Texas Health Science Center Committee for the Protection of Human Subjects.

## 3. Results

### 3.1. Characteristics of Study Participants

Characteristics of decile 1 beneficiaries can be found in Table 1. The study included 155,918 decedent cases and 1,557,481 non-decedent controls. Cases included 4741 Native American and 151,177 non-Native American beneficiaries. Both Native American and non-Native American cases were older than controls. Non-Native American cases had a slightly higher percentage of males, while 52% of Native American cases were female. Cases were also more likely to have dual Medicaid eligibility. The proportion with dual eligibility was highest among Native Americans, including 55.9% of cases and 39.4% of controls, as was the proportion living outside of metropolitan areas (76.4% of cases and 77.3% of controls). Native American beneficiary zip codes had an average of 57–60% Native American residents, while for non-Native American beneficiaries, the average was near 2%. The majority (87%) of Native American beneficiaries lived within tribal lands compared to approximately one in five non-Native Americans. Average annual PM2.5 was slightly higher for cases than controls and lower for Native Americans than non-Native Americans.

### 3.2. Principal Components Analysis

#### 3.2.1. Socioeconomic Position Components

Within decile 1 zip codes, the first component (SEP_PC1)-reflecting socioeconomic disadvantage/material deprivation-accounted for 27.8% of the variance among area SEP indicators (Table S2). Factors with high positive loadings included poverty indicators, unemployment, public assistance, Medicaid coverage, low educational attainment, and household crowding, while factors with strong negative loadings included income, higher educational attainment, and private insurance coverage. The second and third components (SEP_PC2 and SEP_PC3) suggested patterns consistent with housing cost burden and economic inequality, and household and demographic structure, respectively; these explained 9.1% and 8.3% of the variance among area SEP indicators, respectively. SEP_PC1 was retained for logistic regression analysis as it captured the core deprivation gradient within the decile, and there appeared to be a moderate correlation between the three factors among Native Americans (Table S4B). However, an alternative model with SEP_PC2 and SEP_PC2 was included in sensitivity analyses.

#### 3.2.2. Chronic Condition Components

Among decile 1 beneficiaries, the first chronic condition component (CC_PC1)—representing cardiometabolic and vascular disease burden—explained 13.7% of the variance across 27 chronic conditions (Table S3). Component two (CC_PC2), reflecting cognitive decline, explained 5.9% of variance, while component three (CC_PC3), representing conditions associated with aging and longer-term survivorship, explained 5.0% of variance. The fourth component (CC_PC4), representing pulmonary disease burden, explained 4.5% of variance, and component five (CC_PC5), representing cancer, explained 4% of variance. We retained all five chronic condition components as they summarized distinct, non-correlated baseline morbidity factors, and there was an apparent break in the scree plot beginning with the sixth component (Table S3C). However, given that CC_PC1 was the dominant factor, a model excluding CC_PC2–CC_PC5 was included in sensitivity analyses.

#### 3.2.3. Mean PCA Factor Scores by Race

The mean SEP_PC1 score was higher for Native American than non-Native American beneficiaries, while mean scores for SEP_PC2 and SEP_PC3 were higher for non-Native American beneficiaries (Table 2). Cardiometabolic morbidity scores (CC_PC1) were higher among Native American beneficiaries, while all other chronic condition scores were higher among non-Native Americans. Native Americans were slightly less likely to be diagnosed with HTN, IHD, COPD, and asthma compared to non-Native Americans, but more likely to be diagnosed with CHF and CKD, and much more likely to be diagnosed with diabetes (41.0% vs. 19.9%, respectively). Lung cancer diagnoses were rare across both groups (Table 2).

### 3.3. Logistic Regression Analysis

#### 3.3.1. Fully Adjusted Models-Pooled Interaction and Race-Stratified

We first fit fully adjusted pooled and race-stratified (i.e., Native American vs. non-Native American) models to assess heterogeneity in the PM2.5–mortality association by race. In the pooled model, PM2.5 was significantly associated with all-cause mortality among both non-Native American and Native American beneficiaries (OR, 1.01; 95% CI, 1.001–1.02 and OR, 1.12; 95% CI, 1.06–1.18, respectively, Table 3). There was little change among non-Native Americans in stratified models (OR, 1.01; 95% CI, 1.004–1.02); however, the PM2.5 OR was attenuated among Native Americans and no longer significant (OR, 1.05; 95% CI, 0.98–1.13, Table 3).

#### 3.3.2. Sequential Attenuation Models

The sequential attenuation analysis was restricted to Native American beneficiaries. In the base, minimally adjusted model, each 1 µg/m^3^ increase in annual average PM2.5 was associated with a 14% increase in odds of all-cause mortality among Native American beneficiaries (OR, 1.14; 95% CI, 1.09–1.20, Table 4). Adjusting for zip code-level urbanicity resulted in moderate attenuation of the PM2.5 effect (OR, 1.11; 95% CI, 1.06–1.17); however, the inclusion of state fixed effects led to a 57.8% reduction in the PM2.5 OR relative to the base model (OR, 1.06; 95% CI, 0.99–1.13). There was a slight adjustment for area-level SEP, but little impact of individual SEP. Adding chronic disease factors to the model resulted in further attenuation, reducing the base model PM2.5 effect estimate 60.9% (OR, 1.05; 95% CI, 0.98–1.13). The addition of zip code-level proportion of Native American residents slightly increased the PM2.5 risk estimate. Taken together, these results suggest that the increase in PM2.5-related risk among Native Americans observed in the base model was sensitive to adjustment for geographic clustering at the state level and, to a lesser degree, adjustment for area-level SEP and patterns of underlying disease. Results remained consistent in sensitivity analyses including all three SEP principal components (Table S5).

## 4. Discussion

In this study of Medicare beneficiaries residing in low PM2.5-exposed zip codes, we observed a borderline significant association between annual average PM2.5 and all-cause mortality among non-Native American beneficiaries after adjustment for geographic context, socioeconomic position, and baseline morbidity. While the increase was small (OR, 1.01), non-Native American beneficiaries represent 97% of the more than 1.7 million beneficiaries in decile 1. The PM2.5–mortality association was stronger among Native American beneficiaries in minimally adjusted models; however, this effect was attenuated upon adjustment for geographic context, area-level socioeconomic position, and baseline morbidity. Sequential adjustment revealed that state-level population clustering accounted for most of the attenuation of the PM2.5 regression coefficient among Native Americans. When fully adjusted, the PM2.5–mortality association was still elevated but no longer statistically significant.

Geographic context emerged as an important factor in interpreting the PM2.5–mortality relationship among decile 1 Native American beneficiaries. Adjustment for rural–urban continuum resulted in only modest attenuation of the PM2.5 coefficient. In contrast, including state fixed effects—which controlled for differences in baseline mortality across states and estimated the PM2.5–mortality association using exposure variation within, rather than across, states—reduced the PM2.5 risk estimate by nearly 60% relative to the base model. Native American beneficiaries in decile 1 were concentrated in a small number of states, particularly Arizona (32.5%) and New Mexico (27.4%). Although PM2.5 concentrations varied within these states (Table S6), there was little evidence of a PM2.5–mortality association within either population (Table S7), suggesting that the positive PM2.5 coefficient in models without adjustment for geographic context was not explained by strong exposure–response relationships within either of these states. Descriptive analyses also revealed that these two states were among those with comparatively lower mean scores on the chronic conditions components (Table S8), although correlations between PM2.5 exposure and these components were weak (Table S4). In a sensitivity analysis in which state indicators were consolidated to Arizona, New Mexico, and all other states combined (Table S5), the fully adjusted PM2.5–mortality odds ratio decreased from 1.06 (95% CI, 0.99–1.14) to 1.02 (95% CI, 0.95–1.08). These results suggest that in large administrative cohorts with uneven geographic composition, exposure–response estimates are likely sensitive to the specification of geographic variables in regression models.

In addition to geographic clustering, differences in baseline health status also contributed, albeit modestly, to the observed association between PM2.5 and mortality among Native American beneficiaries. When principal components summarizing chronic disease diagnoses were added to the regression models, attenuation of the PM2.5 coefficient increased further, from 59.3% to nearly 61% relative to the base model. Although many of these conditions have been associated with long-term exposure to PM2.5, it is uncertain whether the relatively low concentrations observed in decile 1 zip codes would contribute to the development of chronic disease, although we had no information on past exposure levels. Existing disease may also result in greater susceptibility to PM2.5 exposure [25]. Clarifying whether chronic disease in this population functions primarily as a confounder, mediator, or marker of vulnerability would be an important topic for further research. It is also possible that point source emissions of toxic PM2.5 constituents play a causal role in chronic disease development among U.S. Native American populations that is not reflected by aggregate PM2.5 concentrations [26,27,28,29,30].

Socioeconomic position has frequently been identified as a confounder or effect modifier in air pollution epidemiology studies [10,15,31]. The confounding effect of SEP on the relationship between long-term PM2.5 and mortality has been well described, based on the observation that low-SEP populations generally live in geographic areas with poorer air quality [31,32,33,34]. The challenge in this body of work has been to distinguish the relative impact of low SEP and air pollution and to estimate their joint effect when both factors are present. We investigated the role of SEP as well, but in a context that has not previously been studied; that is, whether SEP confounds or modifies the PM2.5/mortality association even in the presence of very low exposures. Our results indicate that adjustment for either individual- or area-level SEP had relatively little effect on PM2.5-related mortality risk estimates among Native American beneficiaries living in decile 1 zip codes. Dual Medicaid eligibility was the sole individual-level SES variable in the analysis, and our prior work showed that this is a less reliable indicator of poverty among Native Americans than non-Native Americans [16]. The principal component summarizing area-level socioeconomic disadvantage produced only slight additional attenuation beyond that seen after adjustment for geographic context. This result likely reflects the unique exposure profile of this population, in which most Native American beneficiaries, living in tribal areas, were both poorer and exposed to comparatively lower ambient PM2.5 levels. In this setting, socioeconomic disadvantage does not follow the typical pattern of higher-exposure populations, therefore limiting the extent to which SEP acts as a confounder of the PM2.5–mortality relationship.

Finally, community context, represented by the percent of Native Americans residing in the zip code, slightly increased the PM2.5 coefficient when added to the final model; however, it appeared to be health-protective for Native American beneficiaries in models adjusted for geographic context, socioeconomic position, and baseline morbidity. Average chronic condition component scores were relatively low in Arizona and New Mexico—states with extensive tribal lands and the highest concentrations of Native American beneficiaries (Table S8). This is consistent with prior research showing that Native Americans living in rural or tribal communities had lower cigarette and alcohol consumption [35,36] and experienced lower prevalence of certain chronic conditions [37] than those residing in urban settings. It may also reflect benefits of closer proximity to social and cultural support systems [37,38] and/or access to Indian Health Service (IHS) resources [39]. These findings highlight the importance of considering community-level social context when studying environmental health risks in Native American populations.

Our study had several limitations. The accuracy of race coding in the MBSF, particularly for Native American beneficiaries, has been an area of concern [40,41], although this has improved with time due to linkage with IHS records [42]. Second, PM2.5 exposure was estimated only at the residential zip code level, using the zip code centroid concentration, which was then averaged over the preceding 365 days to derive a rolling annual average concentration. While our averaging period was a more precise approach than using a calendar year average, relying on a zip code centroid concentration, although not without precedent [43], may have introduced bias through exposure misclassification. Non-metropolitan areas—where most Native American beneficiaries reside and which generally have lower PM2.5 exposures—also tend to have less fine-scale spatial variability [44]. Additionally, the model used to predict gridded pollutant concentrations performed more poorly in the U.S. “mountain” region (cross-validated R^2^ = 0.769, root mean square error (RMSE) = 3.337; vs. R^2^ = 0.860, RMSE = 2.786 nationally) due to sparse monitoring data and complex terrain [19]. This region includes Arizona and New Mexico, where 60% of decile 1 Native American beneficiaries resided. We also had no estimates of household indoor PM2.5 exposure, which has been shown to exceed outdoor concentrations in tribal lands, primarily due to the use of solid fuels for heating [45,46]. Additionally, person-level covariate information is largely unavailable in the MBSF; thus, area-level SEP measures were used as proxy indicators.

We also acknowledge that a period of 365 days prior to death does not adequately capture chronic PM2.5 exposures, particularly in a cohort ages 65 and older. This is due to both the time-varying nature of air pollution trends and the likelihood of residential mobility. However, we note that the EPA defines short-term and long-term exposure to criteria pollutants as “hours to days” and “months to years”, respectively [47], and that prior Medicare cohort studies evaluating mortality risk from long-term air pollution exposure used calendar-year-specific annual average concentrations for estimating exposure, even in studies with decades-long study periods [5,6,48]. There is also evidence that PM2.5 exposure may not require multiple years to produce an effect. A study of 1-year lagged PM2.5 exposure periods concluded that “the increased risk of death associated with PM2.5 is essentially all manifested within 2 years of exposure” [49]. And finally, MBSF contained data on over 30 million beneficiaries, but our analysis was limited to 1,713,399 subjects living in decile 1 zip codes, including fewer than 156,000 decedent cases. Of these, just 4741 were identified as Native Americans. This imbalance between subgroup sizes likely led to the difference in PM2.5 risk estimates between the pooled and stratified models for Native American beneficiaries, which can influence interaction estimates in pooled regression models [50,51].

However, the Medicare cohort represents the largest and most geographically diverse population of older Americans available for health research in the U.S., which enables studying a relatively small subgroup like Native Americans. While they represent just 3% of decile 1 beneficiaries, Native Americans are the largest racial group in the population of low-PM2.5-exposed individuals in the U.S. aside from the White population. They also represent a sensitive subpopulation, having been subject to significant inequities spanning generations, leading to disproportionately lower SEP and shorter life expectancy [52,53,54].

## 5. Conclusions

In this study, we examine whether socioeconomic and health characteristics could explain the association between low-level PM2.5 exposure and all-cause mortality we observed previously among Native American Medicare beneficiaries [16]. In analyses limited to zip codes within the lowest decile of the U.S. PM2.5 distribution, the observed association was substantially attenuated once adjusted for state-level effects and, to a lesser extent, area SEP and underlying chronic disease patterns. Our findings suggest that the prior observation was influenced in part by geographic clustering of Native American beneficiaries in a small number of states. These findings underscore the importance of accounting for geographic composition, SEP, baseline health status, and community context when interpreting exposure–response relationships in large administrative cohorts, including Medicare, particularly when the focus is on low-exposure populations.

## Figures and Tables

**Table 1:** Characteristics of the study population.

Characteristic	All (n = 1,713,399)		Native American Beneficiaries (n = 44,890)		Non-Native American Beneficiaries (n = 1,668,509)	
	Cases (n = 155,918)	Controls (n = 1,557,481)	Cases (n = 4741)	Controls (n = 40,149)	Cases (n = 151,177)	Controls (n = 1,517,332)
Mean age (yrs)	81.8	74.4	79.1	74.1	81.9	74.4
Sex						
Male	77,968 (50.0%)	752,005 (48.3%)	2276 (48.0%)	17,004 (42.4%)	75,692 (50.1%)	735,001 (48.4%)
Female	77,950 (50.0%)	805,476 (51.7%)	2465 (52.0%)	23,145 (57.7%)	75,485 (49.9%)	782,331 (51.6%)
Race						
White	143,820 (92.2%)	1,419,370 (91.1%)	0 (0.0%)	0 (0.0%)	143,820 (95.1%)	1,419,370 (93.5%)
Black	1640 (1.1%)	15,421 (1.0%)	0 (0.0%)	0 (0.0%)	1640 (1.1%)	15,421 (1.0%)
Asian	1277 (0.8%)	19,095 (1.2%)	0 (0.0%)	0 (0.0%)	1277 (0.8%)	19,095 (1.3%)
Hispanic	2771 (1.8%)	24,489 (1.6%)	0 (0.0%)	0 (0.0%)	2771 (1.8%)	24,489 (1.6%)
Native American	4741 (3.0%)	40,149 (2.6%)	4741 (100%)	40,149 (100%)	0 (0.0%)	0 (0.0%)
Other	939 (0.6%)	11,693 (0.8%)	0 (0.0%)	0 (0.0%)	939 (0.6%)	11,693 (0.8%)
Unknown	730 (0.5%)	27,264 (1.8%)	0 (0.0%)	0 (0.0%)	730 (0.5%)	27,264 (1.8%)
Medicaid-eligible	37,632 (24.1%)	169,839 (10.9%)	2650 (55.9%)	15,806 (39.4%)	34,982 (23.1%)	154,033 (10.2%)
Urbanicity						
Metropolitan	73,252 (47.0%)	745,982 (47.9%)	1117 (23.6%)	9130 (22.7%)	72,135 (47.7%)	736,852 (48.6%)
Micropolitan	37,131 (23.8%)	355,464 (22.8%)	874 (18.4%)	6810 (17.0%)	36,257 (24.0%)	348,654 (23.0%)
Small town	23,042 (14.8%)	220,611 (14.2%)	1286 (27.1%)	11,567 (28.8%)	21,756 (14.4%)	209,044 (13.8%)
Rural	22,467 (14.4%)	235,242 (15.1%)	1464 (30.9%)	12,642 (31.5%)	21,003 (13.9%)	222,600 (14.7%)
Missing	26 (0.0%)	182 (0.0%)	0 (0.0%)	0 (0.0%)	26 (0.0%)	182 (0.0%)
Mean %Native American (zip code-level)	3.74%	3.51%	57.17%	59.89%	2.06%	2.01%
Tribal land indicator	36,628 (23.5%)	353,062 (22.7%)	4084 (86.6%)	34,795 (87.1%)	32,544 (21.5%)	318,267 (21.0%)
Annual PM2.5	3.70	3.69	3.42	3.39	3.71	3.69

**Table 2:** Principal component analysis (PCA) scores and prevalence of select underlying chronic diseases, Native American vs. non-Native American Medicare beneficiaries residing in decile 1 zip codes.

PCA Score/Chronic Disease	Native American Beneficiaries N (%)	Non-Native American Beneficiaries N (%)
Mean SEP_PC1 score	1.976	−0.124
Mean SEP_PC2 score	−0.205	0.466
Mean SEP_PC3 score	−0.635	−0.207
Mean CC_PC1 score (cardiometabolic)	0.059	−0.002
Mean CC_PC2 score (dementia)	−0.029	0.0008
Mean CC_PC3 score (arthritis/aging)	−0.125	0.003
Mean CC_PC4 score (pulmonary)	−0.041	0.001
Mean CC_PC5 score (cancer)	−0.252	0.007
Hypertension	21,135 (47.1%)	742,424 (44.5%)
Congestive heart failure	6544 (14.6%)	210,330 (12.6%)
Ischemic heart disease	9734 (21.7%)	375,271 (22.5%)
Chronic kidney disease	10,590 (23.6%)	289,738 (17.4%)
Diabetes	18,388 (41.0%)	332,182 (19.9%)
Chronic obstructive pulmonary disease	4228 (9.4%)	168,284 (10.1%)
Lung cancer	371 (0.8%)	20,055 (1.2%)
Asthma	2344 (5.2%)	62,761 (3.8%)

**Table 3:** Associations between annual average PM2.5 and all-cause mortality in fully adjusted pooled ^1^ interaction and race-stratified ^2^ models, decile 1 Medicare beneficiaries.

Model	PM2.5 Odds Ratio (95% CI)	*p*-Value
Pooled (Non-Native American)	1.01 (1.001–1.02)	0.03
Pooled (Native American)	1.12 (1.06–1.18)	<0.0001
Stratified Non-Native American	1.01 (1.004–1.02)	0.02
Stratified Native American	1.05 (0.98–1.13)	0.14

^1^ Pooled interaction model included PM2.5, Native American indicator, PM2.5*Native American indicator, age, sex, SEP_PC1, CC_PC1, CC_PC2, CC_PC3, CC_PC4, CC_PC5, dual Medicaid eligibility, RUCA and state. ^2^ Race-stratified model included PM2.5, age, sex, SEP_PC1, CC_PC1, CC_PC2, CC_PC3, CC_PC4, CC_PC5, dual Medicaid eligibility, RUCA, and state.

**Table 4:** Sequential attenuation of the association between annual average PM2.5 and all-cause mortality among Native American Medicare beneficiaries residing in decile 1 zip codes.

Model (Domain Added)	Covariates Added	PM2.5 OR (95% CI)	% Attenuation ^1^ from Base Model
Model 1 (Base Model)	PM2.5 + age + sex	1.14 (1.09–1.20)	--
Model 2 (Geographic Context-urbanicity)	+RUCA	1.11 (1.06–1.17)	19.4%
Model 3 (Geographic Context–state)	+State	1.06 (0.99–1.13)	57.8%
Model 4 (Area-level SEP)	+SEP_PC1	1.06 (0.99–1.12)	59.5%
Model 5 (Person-level SEP)	+Dual	1.06 (0.99–1.13)	59.3%
Model 6 (Baseline Morbidity)	+CC_PC1-CC_PC5	1.05 (0.98–1.13)	60.9%
Model 7 (Community Context)	+%NativeAmerican	1.06 (0.99–1.14)	55.3%

^1^ Percent attenuation was calculated on the log-odds scale using regression coefficients (β) from the maximum likelihood estimates for each model.

## Data Availability

Restrictions apply to the availability of Medicare beneficiary data, which was obtained by the authors from the Centers for Medicare and Medicaid Services, and is subject to a Data Use Agreement.

## Supplementary Materials

The following supporting information can be downloaded at: https://www.mdpi.com/article/10.3390/ijerph23040464/s1. Table S1. AHRQ social determinants of health indicators retained to represent socioeconomic position in principal components analysis; Table S2. Factor loadings, variance explained, and scree plot for socioeconomic position (SEP) principal components; Table S3. Factor loadings, variance explained, and scree plot for chronic condition principal components; Table S4. Correlations between average annual PM25, SEP principal components, and chronic condition principal components, Native American vs. non-Native American Medicare beneficiaries; Table S5. Sensitivity analysis of the PM2.5–mortality association among Native American Medicare beneficiaries using models with alternative SEP and chronic condition principal components and state of residence specification; Table S6. Distribution of annual average PM2.5 concentrations among Native American Medicare beneficiaries residing in decile 1 zip codes, by state; Table S7. Annual average PM2.5–mortality regression estimates and odds ratios among Native American Medicare beneficiaries residing in decile 1 zip codes, by state; Table S8. Mean chronic condition principal component scores among Native American Medicare beneficiaries residing in decile 1 zip codes, by state.

## Abbreviations

The following abbreviations are used in this manuscript:

PM2.5	Fine particulate matter
EPA	The U.S. Environmental Protection Agency
NAAQS	National Ambient Air Quality Standard
SEP	Socioeconomic position
OR	Odds ratio
CI	Confidence interval
CMS	Centers for Medicare and Medicaid Services
MBSF	Master Beneficiary Summary File
AHRQ	Agency for Healthcare Research and Quality
SDOH	Social determinants of health
RUCA	Rural-Urban Commuting Area
PCA	Principal components analysis
IHD	Ischemic heart disease
CHF	Congestive heart failure
CKD	Chronic kidney disease
HTN	Hypertension
COPD	Chronic obstructive pulmonary disease
SEP_PC1	Socioeconomic position principal component one
SEP_PC2	Socioeconomic position principal component two
SEP_PC3	Socioeconomic position principal component three
CC_PC1	Chronic conditions principal component one
CC_PC2	Chronic conditions principal component two
CC_PC3	Chronic conditions principal component three
CC_PC4	Chronic conditions principal component four
CC_PC5	Chronic conditions principal component five
IHS	Indian Health Service
RMSE	Root mean square error

## Appendix A. Regression Models Used in Pooled, Race-Stratified, and Sequential Attenuation Models

(A1)
$$logit\left(P\right)=\beta 0+\beta 1PM+\beta 2NativeAm+\beta 3\left(PM\times NativeAm\right)+\beta 4Age+\beta 5Sex+\beta 6RUCA+\beta 7State\;+\beta 8SEP\_PC1+\beta 9Dual+\beta 10CC\_PC1+\beta 11CC\_PC2+\beta 12CC\_PC3+\beta 13CC\_PC4\;+\beta 14CC\_PC5$$

(A2)
$$logit\left(P\right)=\;\beta 0+\beta 1PM+\beta 2Age+\beta 3Sex+\beta RUCA+\beta 4State+\beta 5SEP\_PC1+\beta 6Dual+\beta 7CC\_PC1\;+\beta 9CC\_PC2+\beta 10CC\_PC3+\beta 11CC\_PC4+\beta 12CC\_PC5$$

(A3)
$$logit\left(P\right)=\beta 0+\beta 1PM+\beta 2Age+\beta 3Sex$$

(A4)
$$logit\left(P\right)=\beta 0+\beta 1PM+\beta 2Age+\beta 3Sex+\beta 4RUCA$$

(A5)
$$logit\left(P\right)=\beta 0+\beta 1PM+\beta 2Age+\beta 3Sex+\beta 4RUCA+\beta 5State$$

(A6)
$$logit\left(P\right)=\beta 0+\beta 1PM+\beta 2Age+\beta 3Sex+\beta 4RUCA+\beta 5State+\beta 6SEP\_PC1$$

(A7)
$$logit\left(P\right)=\beta 0+\beta 1PM+\beta 2Age+\beta 3Sex+\beta 4RUCA+\beta 5State+\beta 6SEP\_PC1+\beta 7Dual$$

(A8)
$$logit\left(P\right)=\beta 0+\beta 1PM+\beta 2Age+\beta 3Sex+\beta 4RUCA+\beta 5State+\beta 6SEP\_PC1+\beta 7Dual+\beta 8CC\_PC1\;+\beta 9CC\_PC2+\beta 10CC\_PC3+\beta 11CC\_PC4+\beta 12CC\_PC5$$

(A9)
$$logit\left(P\right)=\beta 0+\beta 1PM+\beta 2Age+\beta 3Sex+\beta 4RUCA+\beta 5State+\beta 6SEP\_PC1+\beta 7Dual+\beta 8CC\_PC1\;+\beta 9CC\_PC2+\beta 10CC\_PC3+\beta 11CC\_PC4+\beta 12CC\_PC5+\beta 10PctNativeAm$$
