# Supplementary material for: Untangling Impacts of Socioeconomic Position, Chronic Disease, and Low-Level PM2.5 Exposure on Mortality Among Native American Medicare Beneficiaries
Source: Int J Environ Res Public Health. 2026 Apr 4;23(4):464. doi: 10.3390/ijerph23040464 (PMC13116330; doi:10.3390/ijerph23040464)
Supplement: Supplementary file 1 [file ijerph-23-00464-s001.zip › ijerph-4219241-supplementary.pdf]

Supplemental Material

Table S1. AHRQ social determinants of health indicators retained to represent socioeconomic position in principal components analysis

Table S2. Factor loadings, variance explained and scree plot for socioeconomic position (SEP) principal components

Table S3. Factor loadings, variance explained and scree plot for chronic condition principal components

Table S4. Correlations between average annual PM25, SEP principal components, and chronic condition principal components, Native American vs. non-Native American Medicare beneficiaries

Table S5. Sensitivity analysis of the PM2.5-mortality association among Native American Medicare beneficiaries using models with alternative SEP and chronic condition principal components, and state of residence specification

Table S6. Distribution of annual average PM2.5 concentrations among Native American Medicare beneficiaries residing in decile 1 zip codes, by state

Table S7. Annual average PM2.5-mortality regression estimates and odds ratios among Native American Medicare beneficiaries residing in decile 1 zip codes, by state

Table S8. Mean chronic condition principal component scores among Native American Medicare beneficiaries residing in decile 1 zip codes, by state

Table S1. AHRQ social determinants of health indicators retained to represent socioeconomic position in principal components analysis.

Indicator	Indicator Description	Reverse-coded
1. Social Context - Demographics		
ACS_AVG_HH_SIZE_ZC	Average household size	
1. Social Context – Living Conditions		
ACS_PCT_HH_1PERS_ZC	% Single occupant households	
ACS_PCT_CHILD_1FAM_ZC	% Single parent familes	
2. Economic Context - Income		
ACS_GINI_INDEX_ZC	Gini index	
ACS_MEDIAN_HH_INC_ZC	Median household income	yes
2. Economic Context - Poverty		
ACS_PCT_INC50_ZC	% Population w/ income to poverty ratio under 0.5	
ACS_PCT_PERSON_INC_BELOW99_ZC	% Population w/ income to poverty ratio under 1.0	
ACS_PCT_HH_FOOD_STMP_BLW_POV_ZC	% Households below poverty level receiving food stamps	
ACS_PCT_HH_NO_FD_STMP_BLW_POV_ZC	% Households below poverty level not receiving food stamps	
ACS_PCT_HH_FOOD_STMP_ZC	% Households receiving food stamps	
ACS_PCT_HH_PUB_ASSIST_ZC	% Households receiving public assistance or food stamps	
2. Economic Context - Employment		
ACS_PCT_UNEMPLOY_ZC	% Unemployed	
ACS_PCT_NOT_LABOR_ZC	% Not in labor force	
3. Education - Attainment		
ACS_PCT_LT_HS_ZC	% Less than high school education	
ACS_PCT_HS_GRADUATE_ZC	% High school graduates	
ACS_PCT_POSTHS_ED_ZC	% Postsecondary eduction	yes
4. Physical Infrastructure - Housing		
ACS_PCT_HU_BUILT_1979_ZC	% Housing units built before 1979	
ACS_PCT_HU_KITCHEN_ZC	% Housing units lacking complete kitchen facilities	
ACS_PCT_HU_MOBILE_HOME_ZC	% Mobile home housing units	
ACS_PCT_HU_PLUMBING_ZC	% Housing units lacking complete plumbing facilities	

ACS_PCT_OWNER_HU_COST_50PCT_ZC	% Owner-occupied housing w/ costs ≥50% of household income	
ACS_PCT_OWNER_HU_COST_30PCT_ZC	% Owner-occupied housing w/ costs ≥30% of household income	
ACS_PCT_OWNER_HU_ZC	% Owner occupied housing	yes
ACS_PCT_RENTER_HU_COST_30PCT_ZC	% Renter-occupied housing w/ rent ≥30% of household income	
ACS_PCT_RENTER_HU_COST_50PCT_ZC	% Renter-occupied housing w/ rent ≥50% of household income	
ACS_PCT_VACANT_HU_ZC	% Vacant housing	
ACS_PCT_1UP_PERS_1ROOM_ZC	% Housing units w/ >1 occupant per room	
4. Physical Infrastructure - Transportation		
ACS_PCT_HU_NO_VEH_ZC	% Housing units w/ no vehicle	
ACS_PCT_WORK_NO_CAR_ZC	% Workers in households w/ no vehicle	
5. Healthcare Context - Health Insurance Status		
ACS_PCT_MEDICAID_ANY_ZC	% Population w/ Medicaid health insurance	
ACS_PCT_PRIVATE_SELF_ZC	% Population w/ direct-purchase health insurance	yes
ACS_PCT_PRIVATE_EMPL_ZC	% Population w/ employer-based health insurance	yes
ACS_PCT_PRIVATE_ANY_ZC	% Population w/ any private health insurance	yes
ACS_PCT_UNINSURED_ZC	% Population uninsured	

Table S2. Factor loadings, variance explained and scree plot for socioeconomic position (SEP) principal components.

A. Factor Loadings

Variable	PC1	PC2	PC3
% Population w/ income to poverty ratio under 1.0	**0.86**	0.17	0.02
% Households below poverty level receiving food stamps	**0.84**	0.09	-0.19
% Households receiving food stamps	**0.83**	0.05	-0.24
% Households receiving public assistance or food stamps	**0.83**	0.06	-0.24
% Population w/ Medicaid health insurance	**0.81**	0.03	-0.14
% Population w/ income to poverty ratio under 0.5	**0.71**	0.23	-0.04
% Less than high school education	**0.66**	-0.16	-0.16
% Unemployed	**0.61**	0.15	-0.15
% Population uninsured	**0.59**	-0.06	-0.03
% Housing units w/ >1 occupant per room	**0.58**	0.01	-0.38
% Single parent familes	**0.49**	0.18	-0.03
% Not in labor force	**0.47**	-0.10	0.38
% Households below poverty level not receiving food stamps	**0.45**	0.17	0.33
% Mobile home housing units	**0.43**	-0.33	-0.004
% Housing units lacking complete kitchen facilities	**0.40**	-0.42	0.34
% Housing units lacking complete plumbing facilities	**0.40**	**-0.46**	0.34
% Housing units w/ no vehicle	0.35	**0.51**	0.05
Average household size	0.34	-0.15	**-0.70**
% High school graduates	0.31	**-0.58**	0.14
Gini index	0.26	**0.44**	0.31
% Renter-occupied housing w/ rent ≥30% of household income	0.17	0.38	0.08
% Renter-occupied housing w/ rent ≥50% of household income	0.16	0.36	0.13
% Workers in households w/ no vehicle	0.14	**0.47**	0.05
% Vacant housing	0.11	-0.18	**0.60**
% Single occupant households	0.10	0.29	**0.67**

% Owner-occupied housing w/ costs ≥50% of household income	0.04	**0.47**	0.18
% Housing units built before 1979	0.01	-0.05	0.29
% Owner-occupied housing w/ costs ≥30% of household income	-0.02	**0.48**	0.08
% Owner occupied housing	-0.30	**-0.48**	0.12
% Population w/ direct-purchase health insurance	-0.34	-0.01	0.30
% Postsecondary eduction	**-0.61**	**0.52**	-0.01
% Population w/ employer-based health insurance	**-0.68**	0.06	-0.38
Median household income	**-0.71**	0.14	-0.39
% Population w/ any private health insurance	**-0.88**	0.04	-0.05

B. Variance Summary

Principal Component	Eigenvalue	Variance Explained	Cumulative Variance Explained
SEP_PC1	9.46	27.8%	27.8%
SEP_PC2	3.11	9.1%	37.0%
SEP_PC3	2.83	8.3%	45.3%

C. Scree Plot

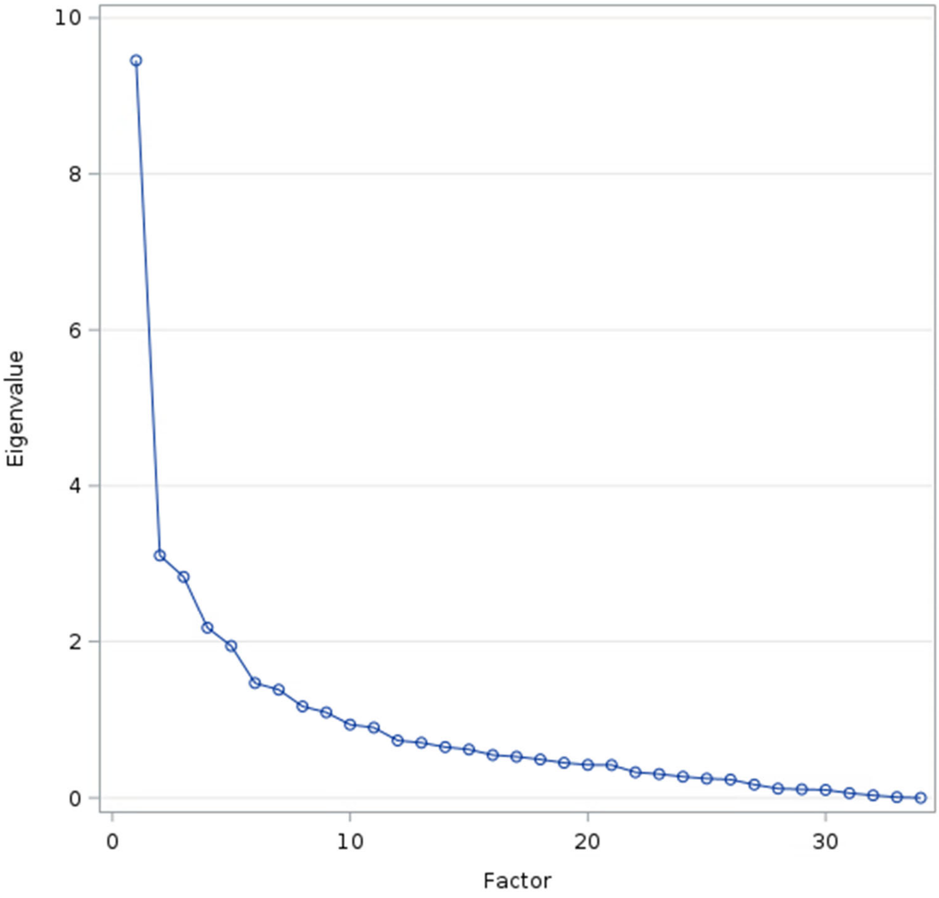

Table S3. Factor loadings, variance explained and scree plot for chronic condition principal components.

A. Factor Loadings

Variable	PC1	PC2	PC3	PC4	PC5
Hypertension	**0.66**	-0.22	0.12	-0.23	-0.02
Congestive heart failure	**0.61**	0.02	-0.29	0.21	-0.14
Chronic kidney disease	**0.61**	-0.05	-0.18	-0.04	-0.17
Ischemic heart disease	**0.59**	-0.14	-0.25	-0.003	-0.06
Anemia	**0.57**	0.03	0.004	0.14	0.03
Hyperlipidemia	**0.53**	-0.34	0.16	-0.31	0.002
Chronic obstructive pulmonary disease	**0.45**	-0.03	-0.05	**0.47**	0.18
Diabetes	**0.44**	-0.20	-0.12	-0.26	-0.30
Atrial fibrillation	**0.42**	-0.06	-0.18	0.12	-0.02
Alzheimer's or other dementia	**0.41**	**0.74**	-0.06	-0.16	0.04
Depression	0.37	0.20	0.28	0.02	0.07
Rheumatoid arthritis	0.37	-0.05	0.37	-0.07	0.14
Hypothyroidism	0.33	-0.01	0.37	-0.08	-0.15
Stroke	0.28	0.07	-0.04	-0.07	-0.04
Alzheimer's	0.25	**0.77**	-0.03	-0.24	0.06
Benign prostatic hyperplasia	0.25	-0.11	-0.21	-0.18	**0.46**
Acute myocardial infarction	0.24	-0.06	-0.24	0.12	-0.20
Asthma	0.22	-0.09	0.21	0.30	0.15
Osteoporosis	0.20	0.12	**0.48**	0.19	-0.01
Hip fracture	0.19	0.24	0.13	0.16	0.12
Lung cancer	0.14	-0.04	-0.03	**0.47**	0.25
Colorectal cancer	0.12	-0.02	-0.03	0.14	0.13
Prostate cancer	0.12	-0.11	-0.16	-0.16	**0.51**
Breast cancer	0.09	-0.004	0.37	0.19	-0.27
Glaucoma	0.06	-0.13	0.19	-0.19	0.07

Endometrial cancer	0.05	-0.01	0.12	0.10	-0.26
Cataract	0.05	-0.19	0.31	-0.18	0.29

B. Variance Summary

Principal Component	Eigenvalue	Variance Explained	Cumulative Variance Explained
CC_PC1	3.71	13.7%	13.7%
CC_PC2	1.58	5.9%	19.6%
CC_PC3	1.34	5.0%	24.6%
CC_PC4	1.20	4.5%	29.0%
CC_PC5	1.08	4.0%	33.0%

C. Scree Plot

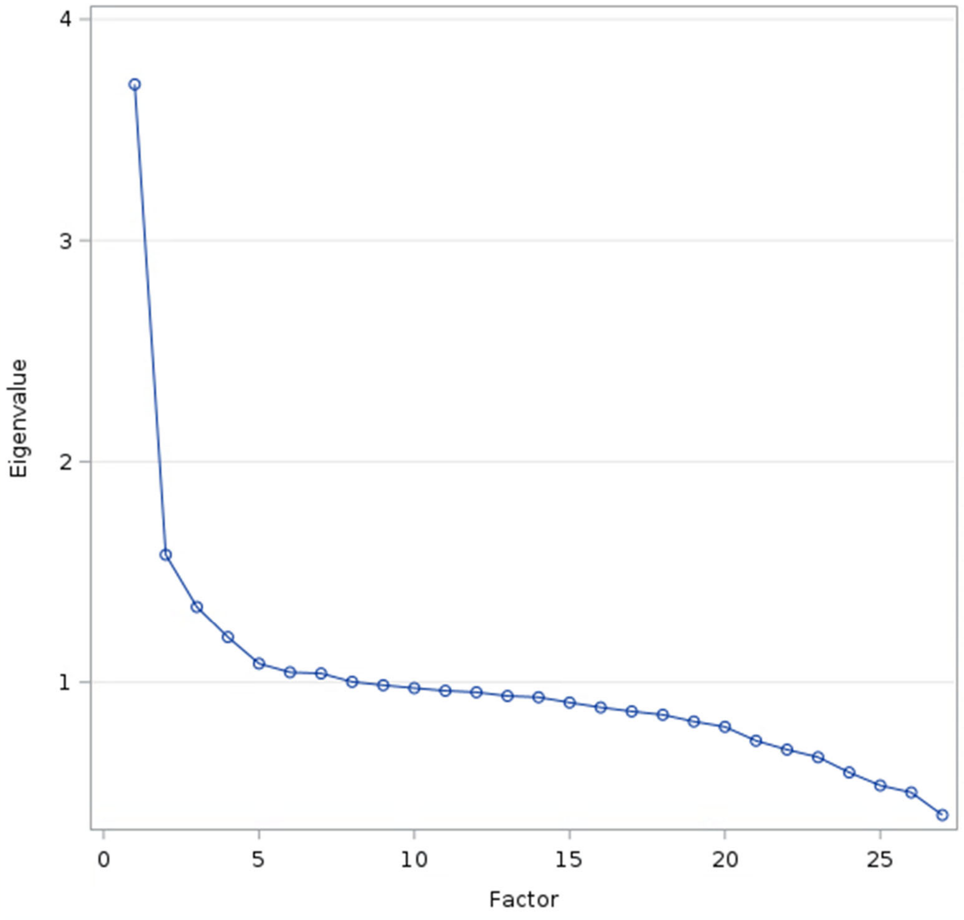

Table S4. Correlations between average annual PM25, SEP principal components, and chronic condition principal components, Native American vs. non-Native American Medicare beneficiaries.

A. Non-Native American beneficiaries

	PM2.5	SEP_PC1	SEP_PC2	SEP_PC3	CC_PC1	CC_PC2	CC_PC3	CC_PC4	CC_PC5
PM2.5	1.00	-0.06	0.05	-0.10	0.05	-0.002	0.008	-0.015	-0.003
SEP_PC1	-0.06	1.00	-0.07	0.16	0.04	-0.009	-0.04	0.02	-0.04
SEP_PC2	0.05	0.05	-0.07	0.15	-0.01	0.01	0.04	-0.01	0.02
SEP_PC3	-0.10	0.16	0.15	1.00	-0.004	-0.007	0.01	0.01	0.03
CC_PC1	0.05	0.04	-0.01	-0.004	1.00	0.002	0.0009	0.001	0.004
CC_PC2	-0.002	-0.009	0.01	-0.007	0.002	1.00	-0.002	-0.001	-0.002
CC_PC3	0.008	-0.04	0.04	0.01	0.0009	-0.002	1.00	-0.0003	-0.004
CC_PC4	-0.015	0.02	-0.01	0.01	0.001	-0.001	-0.0003	1.00	-0.003
CC_PC5	-0.003	-0.04	0.02	0.03	0.004	-0.002	-0.004	-0.003	1.00

B. Native American beneficiaries

	PM2.5	SEP_PC1	SEP_PC2	SEP_PC3	CC_PC1	CC_PC2	CC_PC3	CC_PC4	CC_PC5
PM2.5	1.00	-0.35	0.34	0.06	0.06	-0.01	-0.02	0.06	0.01
SEP_PC1	-0.35	1.00	-0.39	-0.31	-0.03	-0.02	-0.01	-0.04	-0.05
SEP_PC2	0.34	-0.39	1.00	-0.17	0.06	0.009	-0.03	0.07	-0.002
SEP_PC3	0.06	-0.31	-0.17	1.00	-0.03	0.03	0.03	0.002	0.05
CC_PC1	0.06	-0.03	0.06	-0.03	1.00	-0.06	-0.03	-0.04	-0.14
CC_PC2	-0.01	-0.02	0.009	0.03	-0.06	1.00	0.06	0.04	0.10
CC_PC3	-0.02	-0.01	-0.03	0.03	-0.03	0.06	1.00	0.006	0.13
CC_PC4	0.06	-0.04	0.07	0.002	-0.04	0.04	0.006	1.00	0.11
CC_PC5	0.01	-0.05	-0.002	0.05	-0.144	0.10	0.13	0.11	1.00

Table S5. Sensitivity analysis of the PM2.5-mortality association among Native American Medicare beneficiaries using models with alternative SEP and chronic condition principal components, and state of residence specification.

Model specification	PM2.5 β	Odds ratio	95% CI
Fully adjusted model	0.0594	1.06	0.99-1.14
Inclusion of SEP_PC2 and SEP_PC3	0.0592	1.06	0.99-1.14
Inclusion of only CC_PC1	0.0416	1.04	0.97-1.12
State of residence specified as AZ/NM//Other	0.0152	1.02	0.95-1.08

Table S6. Distribution of annual average PM2.5 concentrations among Native American Medicare beneficiaries residing in decile 1 zip codes, by state.

State	N	Mean (STD)	Minimun	25th Pctl	75th Pctl	Maximun
All	44,890	3.39 (0.64)	0.54	2.88	3.86	4.68
Arizona	14,609	3.01 (0.41)	0.70	2.75	3.20	4.68
California	2,867	3.82 (0.60)	1.33	3.47	3.90	4.68
Colorado	449	3.15 (0.64)	1.61	2.72	3.58	4.68
Florida	31	4.16 (0.38)	3.46	3.82	4.52	4.68
Idaho	280	4.02 (0.60)	1.93	3.69	4.52	4.68
Maine	321	3.69 (0.61)	2.69	3.11	4.36	4.68
Massachusetts	25	4.52 (0.16)	4.10	4.44	4.64	4.67
Michigan	356	4.17 (0.37)	3.02	3.94	4.47	4.68
Minnesota	1,088	4.08 (0.52)	2.48	3.83	4.27	4.68
Montana	1,741	3.91 (0.56)	2.06	3.60	4.36	4.68
Nebraska	109	3.85 (0.49)	2.71	3.57	4.26	4.68
Nevada	981	3.22 (0.61)	0.54	2.82	3.63	4.65
New Hampshire	11	4.37 (0.32)	3.75	4.31	4.61	4.66
New Mexico	12,304	3.31 (0.53)	1.88	2.92	3.60	4.68
New York	341	4.42 (0.15)	3.69	4.34	4.51	4.68
North Carolina	613	4.46 (0.14)	4.09	4.37	4.57	4.68
North Dakota	907	4.00 (0.50)	2.63	3.52	4.44	4.68
Oregon	886	3.65 (0.67)	1.45	3.27	4.23	4.67
Rhode Island	21	4.18 (0.44)	3.45	3.80	4.57	4.65
South Dakota	2,836	3.72 (0.58)	2.03	3.32	4.19	4.68
Texas	43	4.17 (0.50)	2.58	4.08	4.54	4.66
Utah	850	3.25 (0.65)	1.96	2.68	3.73	4.68
Washington	2,107	3.60 (0.76)	1.52	3.03	4.22	4.68
Wisconsin	313	4.28 (0.32)	3.02	4.11	4.53	4.68
Wyoming	776	3.37 (0.50)	1.99	3.06	3.68	4.58

Table S7. Annual average PM2.5-mortality regression estimates and odds ratios among Native American Medicare beneficiaries residing in decile 1 zip codes, by state.

State	N	PM2.5 β	SE	OR (95% CI)	p-value
Arizona	14,609	-0.0213	0.0834	0.98 (0.83-1.15)	0.80
California	2,867	-0.1035	0.1177	0.90 (0.72-1.14)	0.38
Colorado	449	-0.8340	0.3787	0.43 (0.21-0.91)	0.03
Idaho	280	0.6519	0.4738	1.92 (0.76-4.86)	0.17
Maine	321	0.6739	0.7071	1.96 (0.49-7.84)	0.34
Michigan	356	0.3374	0.7256	1.40 (0.34-5.81)	0.64
Minnesota	1,088	-0.0701	0.2583	0.93 (0.56-1.55)	0.79
Montana	1,741	0.2246	0.1654	1.25 (0.91-1.73)	0.17
Nebraska	109	-0.7334	0.8657	0.48 (0.09-2.62)	0.40
Nevada	981	-0.2280	0.2530	0.80 (0.49-1.31)	0.37
New Mexico	12,304	0.0097	0.0757	1.01 (0.87-1.17)	0.90
New York	341	-1.2625	1.8169	0.28 (0.01-9.96)	0.49
North Carolina	613	0.1904	1.0022	1.21 (0.17-8.63)	0.85
North Dakota	907	-0.0578	0.2569	0.94 (0.57-1.56)	0.82
Oregon	886	0.2071	0.2247	1.23 (0.79-1.91)	0.36
South Dakota	2,836	0.1091	0.1240	1.12 (0.88-1.420	0.38
Texas	43	0.5918	2.6363	1.81 (0.01-317.00)	0.82
Utah	850	0.3304	0.2265	1.39 (0.89-2.17)	0.14
Washington	2,107	0.1006	0.1050	1.11 (0.90-1.36)	0.34
Wisconsin	313	-0.0756	0.7391	0.93 (0.22-3.95)	0.92
Wyoming	776	0.4907	0.3711	1.63 (0.79-3.38)	0.19

Table S8. Mean chronic condition principal component scores [1] among Native American Medicare beneficiaries residing in decile 1 zip codes, by state.

State	Mean CC_PC1	Mean CC_PC2	Mean CC_PC3	Mean CC_PC4	Mean CC_PC5
All	0.0588	-0.0294	-0.1252	-0.0408	-0.2517
Arizona	0.0039	-0.0173	-0.0936	-0.1340	-0.2734
California	0.2032	-0.073	-0.1639	0.0276	-0.2407
Colorado	0.0392	0.0960	-0.1456	-0.0026	-0.2599
Florida	0.3773	-0.3905	0.1827	0.0828	-0.7851
Idaho	0.0326	-0.0919	-0.0080	-0.0390	-0.1778
Maine	0.3513	-0.0808	-0.1048	0.2116	-0.1859
Massachusetts	-0.0664	-0.0304	0.0103	-0.1562	-0.0045
Michigan	0.2731	0.0180	-0.0076	0.1297	-0.0075
Minnesota	0.2571	-0.0420	-0.2835	0.1721	-0.2239
Montana	0.1115	-0.0064	-0.1511	0.1734	-0.2067
Nebraska	0.1212	0.1272	-0.0164	0.1873	-0.0802
Nevada	0.0554	-0.0450	-0.0824	-0.0558	-0.0227
New Hampshire	0.0456	-0.1243	0.1829	0.2365	-0.1691
New Mexico	-0.0036	-0.0342	-0.0763	-0.1436	-0.2552
New York	-0.2286	-0.0427	-0.3303	0.0344	-0.1574
North Carolina	0.3874	-0.0113	-0.1865	-0.1420	-0.4286
North Dakota	0.1812	-0.1342	-0.3041	0.1610	-0.3145
Oregon	0.0613	0.0002	-0.1337	0.1680	-0.2185
Rhode Island	0.2933	-0.3748	-0.0605	0.2231	-0.1826
South Dakota	0.2158	-0.0576	-0.2325	0.2020	-0.2705
Texas	0.2890	0.2167	0.1423	-0.1978	0.0253
Utah	-0.0778	0.0504	-0.1655	-0.0634	-0.2337
Washington	0.1246	-0.0277	-0.1486	0.1663	-0.1242
Wisconsin	0.3060	-0.0884	-0.3543	0.1081	-0.4006
Wyoming	-0.0639	0.0391	-0.3469	0.1712	-0.3041

[1] CC_PC1, cardiometabolic and vascular disease; CC_PC2, cognitive decline/dementia; CC_PC3, arthritis/aging; CC_PC4, pulmonary disease; CC_PC5, cancer.
